# Phomoxanthone A, Compound of Endophytic Fungi *Paecilomyces* sp. and Its Potential Antimicrobial and Antiparasitic

**DOI:** 10.3390/antibiotics11101332

**Published:** 2022-09-29

**Authors:** Gisele da Costa Ramos, João Victor Silva-Silva, Luciano Almeida Watanabe, José Edson de Sousa Siqueira, Fernando Almeida-Souza, Kátia S. Calabrese, Andrey Moacir do Rosario Marinho, Patrícia Santana Barbosa Marinho, Aldo Sena de Oliveira

**Affiliations:** 1 Post-Graduate Program in Chemistry, Federal University of Pará, Belém 66075-110, Brazil; 2 Laboratory of Immunomodulation and Protozoology, Oswaldo Cruz Institute, Oswaldo Cruz Foundation, Rio de Janeiro 81310-020, Brazil; 3 Laboratory of Medicinal and Computational Chemistry, Institute of Physics of São Carlos, University of São Paulo, São Carlos 13418-900, Brazil; 4 Post-Graduate in Animal Sciences, State University of Maranhão, São Luís 65690-000, Brazil; 5 Department of Exact Sciences and Education, Federal University of Santa Catarina, Blumenau 88040-900, Brazil

**Keywords:** phomoxanthone A, *Bacillus subtilis*, *Leishmania amazonensis*, *Trypanosoma cruzi*, S-ribosyl-homocysteine lyase, computational studies

## Abstract

The present work reports the isolation and biological evaluation of three dimeric xanthones from *Paecilomyces* sp. EJC01.1 isolated as endophytic from *Schnella splendens*, a typical plant of the Amazon. The compounds phomoxanthone A (**1**), phomoxanthone B (**2**) and dicerandrol B (**3**) were isolated by chromatographic procedures and identified by spectroscopic methods of 1D and 2D NMR and MS. The extracts and compound **1** showed antimicrobial activities against *Bacillus subtilis*, *Escherichia coli*, *Staphylococcus aureus*, *Salmonella typhimurium* and *Pseudomonas aeruginosa*. The compound phomoxanthone A (**1**) showed greater inhibitory activity against *B. subtilis* (MIC of 7.81 µg mL^−1^); in addition, it also pronounced inhibitory effect against promastigote forms of *Leishmania amazonensis* (IC_50_ of 16.38 ± 1.079 µg mL^−1^) and epimastigote forms *Trypanosoma cruzi* (IC_50_ of 28.61 ± 1.071 µg mL^−1^). To provide more information about the antibacterial activity of compound 1, an unprecedented molecular docking study was performed using S-ribosyl-homocysteine lyase (LuxS) (PDB ID 2FQO), which showed a possible interaction of phomoxanthone A with two of the residues (His58 and Cys126) that are fundamental for the catalysis mechanism in *B. subtilis*, which may be associated with the higher activity, when compared to other bacteria, observed in experimental studies. Additionally, quantum studies (DFT) were performed, for which a low gap value (5.982 eV) was observed, which corroborates the reactivity of phomoxanthone A. Thus, phomoxanthone A can be a good agent against pathogenic bacteria.

## 1. Introduction

Species from *Paecilomyces* genus can produce a variety of secondary metabolites of different chemical classes and with varied biological activities, such as antimicrobial, antiviral, antitumor, herbicidal, insecticidal, antiplasmodial, antitrypanosomal, nematicidal, cytotoxic, enzyme inhibitors, phytotoxicity and radical scavenging, some of which are important in the pharmaceutical and agrochemical markets [1,2].

Some groups of substances are produced by fungi from genus *Paecilomyces*, such as terpenoids and steroids, peptides including diketopiperazines, alkaloids and other nitrogen-containing compounds, shikimate-derived metabolites and lipids, and polyketides [3]. Polyketides, which are a major class of natural products, possess several pharmacological properties, including xanthones, among their derivatives due to their biosynthetic precursor [4,5] and can be isolated from plants and microorganisms, including endophytic [6,7]. Although of simple chemical structure, the xanthones have shown many different chemical substituents generating a large kind of the xanthones. The xanthones isolated so far may be classified into five major groups: simple oxygenated xanthones, xanthone glycosides, prenylated and related xanthones, xanthonolignoids, and miscellaneous xanthones [8]. The xanthones have showed a large variety of biological activities, such as: anticancer, antimicrobial, antimalarial, anticholinesterase, anti-HIV, anti-inflammatory, anticonvulsant, antioxidant, and cardiovascular properties [9]. Dimeric xanthones from an endophytic fungi *Phomopsis* sp. BCC 1323 and *Aschersonia* sp. BCC 8401 demonstrated antimalarial actions [10,11] and varied biological activities [12,13], including antimicrobials and anticancer as dicerandrol C [14], antimalarials as ascherxanthone A [11] and mycotoxins as secalonic acids B and D [15,16].

**Figure 1:**
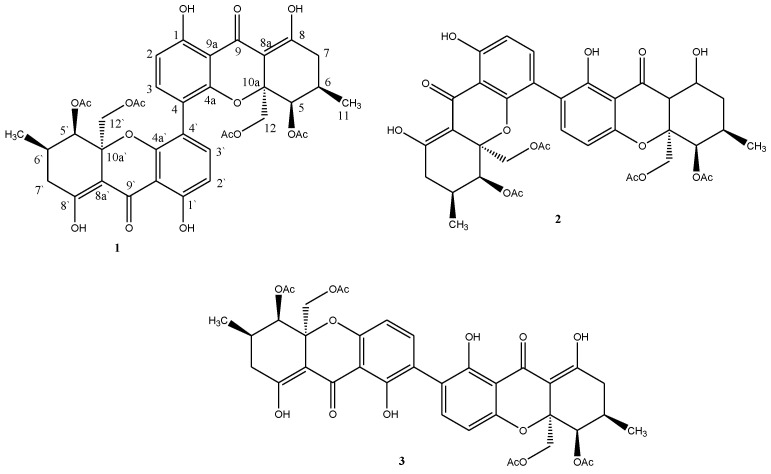
Dimeric xanthones isolated from *Paecilomyces* sp. EJC01.1.

In this context, the chemistry study of fungus *Paecilomyces* sp. EJC01.1 isolated of *Schnella splendens*, a typical Amazon plant was performed. This work evaluated the antimicrobial and antiprotozoal activities of the major compound Phomoxanthone A (**1**) isolated from organic extracts and we are also reporting the first occurrence of dimeric xanthone from genus *Paecilomyces* (Figure 1).

In addition to experimental studies, we report in this article the use of computational strategies for data acquisition and investigation of possible molecular mechanisms involved in biological activity. The integration of experimental and computational methods is of enormous importance in the identification and development of new bioactive molecules from collections of real or virtual compounds. In this context, the SBDD (structure-based drug design) and LBDD (ligand-based drug design) strategies are naturally highlighted [17]. SBDD methods are based on the knowledge of the topological arrangement of biological targets; therefore, they necessarily use detailed 3D data of the macromolecule under study. This information can be obtained by analyzing crystallographic structures, NMR or homology modeling.

In this article, we use molecular docking, one of the main strategies of SBDD, which consists of predicting the bioactive conformation of a small molecule (ligand) at the binding site of a macromolecule (target protein), followed by evaluation (scoring) and classification of the proposed connection mode. In addition to molecular docking studies, we report the use of density functional theory (DFT), used to calculate the energy referring to the surfaces of the HOMO (highest occupied molecular orbital), LUMO (lowest unoccupied molecular orbital) and the gap (difference between the orbital energies), which were considered important chemical reactivity indicators, especially for phomoxanthone A (**1**), the most active compound in antibacterial assays.

## 2. Results

### 2.1. Isolation and Identification of Chemical Constituents

Compounds **1**–**3** were obtained from the chromatographic fractionation of the ethyl acetate fraction, obtained from the extract of *Paecilomyces* sp. EJC01.1.

The ^1^H NMR to **1** showed the signals at δ 6.58 (*d*, 8.7, H-2) and δ 7.38 (*d*, 8.7, H-3) that allowed propose an aromatic ring with *ortho*-related hydrogen. The signals at δ 11.50 and δ 14.09 are characteristic to hydroxyl chelated at carbonyl group. The signal at δ 11.50 showed HMBC correlation with the carbon signal at δ 161.6 (C-1), and H-3 also showed HMBC correlation with C-1 and δ 153.8 (C-4a) signal; these data allowed proposing a tetra-replaced aromatic ring. In the ^1^H NMR to **1** is still observed the signals at δ 4.28 (*d*, 12.6) e δ 4.17 (*d*, 12.9), these signals showed HSQC correlation with the carbon signal at δ 64.5 (C-12) evidencing diastereotopic hydrogens. The diastereotopic hydrogens H-12a and H-12b showed HMBC correlation with the signal at δ 170.0 attributed to carbonyl group of the acetate and the signal δ 100.1 attributed to C-8a. The signals at δ 2.46 (*m*) and δ 2.33 (*m*) were attributed the hydrogens H-7a and H-7b and showed HMBC correlation with the signals δ 100.1 (C-8a) and δ 177.6. The chelated hydrogen at δ 14.09 also showed HMBC correlation with δ 177.6, allowing it to be assigned to 8-OH and δ 177.6 to C-8a. Still, it was observed at δ 1.01 (*d*, 6.0) assigned to methyl and sign at δ 2.07 (*s*) assigned to another acetate group in the molecule. HMBC correlations analysis suggested the presence of a tetrahydroxanthone unit. However, the MS spectrum of **1** (ESI) showed *m*/*z* 773 [M + Na]^+^, which indicated a molecular mass of 750 Da. These data were decisive for structural determination of **1**, because the presence of only 19 carbon signals in the ^13^C NMR spectrum indicated the symmetry of this compound with molecule formula C_38_H_38_O_16_. Through spectral analysis and by comparison with previously reported data [10], **1** was determined as the phomoxanthone A (Figure 1).

The MS spectrum of **2** (ESI) showed that *m/z* 773 [M + Na]^+^ indicated the same molecular formula as phomoxanthone A (**1**), C_38_H_38_O_16_. However, analyses of 1D and 2D NMR spectra revealed that **2** is not symmetrical being also a dimer of the same monomeric xanthone unit as in **1**, however, with the dimeric bonding between C-2 and C-4′. The spectral data of **2** were consistent with those described in the literature for phomoxanthone B [10] (Figure 1).

The ^1^H and ^13^C NMR spectra of **3** indicated structural similarity to both compounds **1** and **2**. The MS spectrum (ESI) *m*/*z* 707 [M-H]^−^ indicates a molecular mass of 708 Da, consistent with the molecular mass of dicerandrol B (Figure 1), previously isolated from *Phomopsis longicola* [18].

The compound **1** was the majority compound isolated from these series, enabling in vitro antimicrobial and antiprotozoal evaluation.

### 2.2. Antimicrobial Activity

The hexanic, ethyl acetate, and methanol extracts were tested against *Bacillus subtilis*, *Escherichia coli* and *Pseudomonas aeruginosa*. The ethyl acetate extract displayed better results against *E. coli* and *B. subtilis* up to 39.06 µg mL^−1^ (Table 1). Then, the ethyl acetate extract was fractioned on silica gel column chromatography, leading to the isolation of the bioactive compounds **1**–**3**. The compound **1** obtained in greater quantity (59.5 mg) from ethyl acetate extract of *Paecilomyces* sp. EJC01.1 was phomoxanthone A (**1**), and for that reason the substance was evaluated for antimicrobial activity (Table 1).

Phomoxanthone A (**1**) was active with a bactericidal effect against *B. subtilis* up to the concentration of 7.81 µg mL^−1^ and inhibited the growth of *E. coli* and *Staphylococcus aureus*, presenting a bacteriostatic effect of up to concentrations of 125 and 500 µg mL^−1^, respectively.

### 2.3. In Vitro Antiprotozoal

The compound phomoxanthone A (**1**) was selected to assess its leishmanicidal and trypanocidal potential. The phomoxanthone A (**1**) was active against promastigote forms of *L. amazonensis* (IC_50_ 16.38 µg mL^−1^) and *T. cruzi* (IC_50_ 28.61 µg mL^−1^). In the cytotoxicity assay, the compound **1** showed CC_50_ of the 28.79 µg mL^−1^ giving SI of the 1.01 to *T. cruzi* and SI of the 1.76 to *L. amazonensis*. The results showed phomoxanthone A is more active and selective to *L. amazonensis* then *T. cruzi* (Table 2).

### 2.4. Computational Studies

#### 2.4.1. Molecular Docking

**Figure 2:**
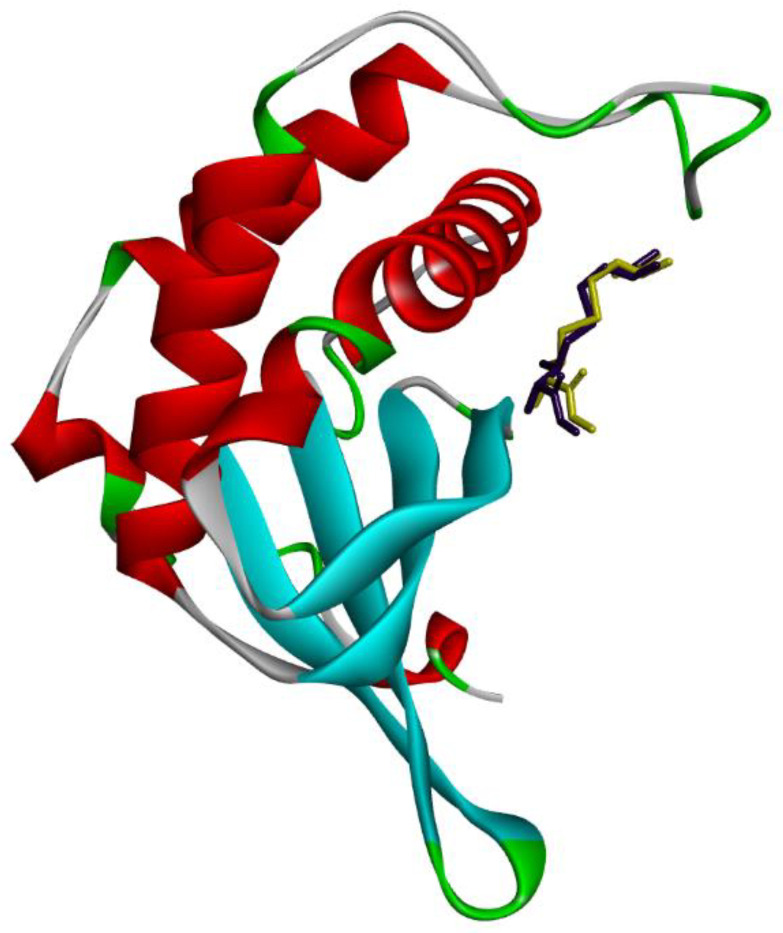
Redocking of the (2s)-2-amino-4-[(2r,3r)-2,3-dihydroxy-3-n-hydroxycarbamoyl-propylmercapto] butyric acid ligand at the LuxS active site (PDB ID 2FQO). In yellow, the best pose of the crystallized ligand generated by the GoldScore function, and, in lilac, its crystallographic conformation. The beta sheets of the protein are represented in cyan blue and the alpha helices in red. The figure was generated by the Biovia Discovery Studio Visualizer software (v19.1.0.18287, BIOVIA, San Diego, CA, USA).

To carry out the computational studies, we only used data obtained in antibacterial studies, presented in Table 1. Therefore, molecular docking studies were carried out for which the four functions available in the GOLD program were used (ASP, ChemScore, GoldScore and ChemPLP). Redocking studies (Figure 2) were performed to evaluate the robustness of the applied methodology, for which the cocrystallized ligand pose and the RMSD (root mean square deviation) values were evaluated. The GoldScore function, which predicts ligand bond positions considering factors, such as hydrogen bonding energy, van der Waals energy, metal interaction and torsional stress, that were chosen because of its high degree of alignment and low RMSD value (0.9136 Å).

**Figure 3:**
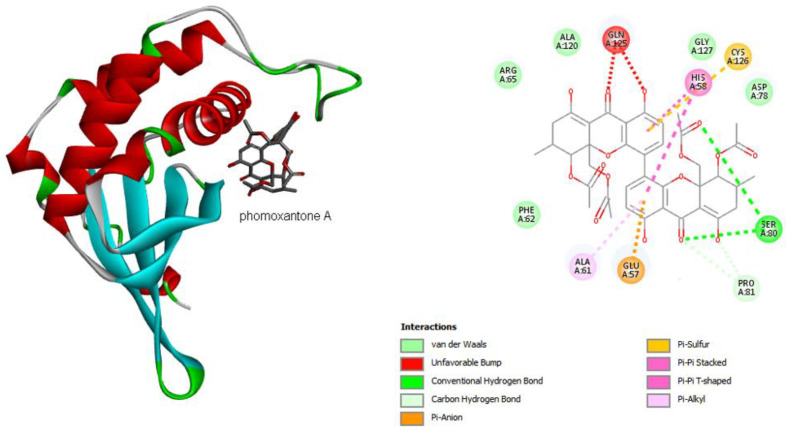
The 3D (**left**) and 2D (**right**) diagrams showing the interactions of phomoxanthone A at the catalytic site of LuxS (PD ID 2FQO). The beta sheets of the protein are represented in cyan blue and the alpha helices in red. The carbon atoms of phomoxanthone A are represented in gray, the oxygen atoms in red, and the hydrogen atoms were suppressed for better visualization of the image. The figure was generated by the Biovia Discovery Studio Visualizer software (v19.1.0.18287, BIOVIA, San Diego, CA, USA).

Analyzing the interactions between phomoxanthone A and the residues present in the catalytic site of LuxS (Figure 3), it is possible to observe a diversity of interactions, for which it is worth mentioning: a Pi–sulfur type interaction between Cys126 and one of the aromatic systems of phomoxanthone A and with the residue His58 two interactions, one of them of the Pi-Pi-stacked type and the other Pi-Pi-shaped, one with each aromatic ring of the phomoxanthone A. It is important to mention that LuxS is a homodimeric metalloenzyme with two identical active sites, each with a metal ion that acts as a cofactor. Initially, it was proposed that the ion would be zinc (Zn^2+^); however, it has been shown that the enzymatic activity is superior with bivalent iron, which is currently accepted. The ion is coordinated with three residues (in *Bacillus subtilis*, they are His54, His58 and Cys126) [19,20], among which two establish direct interactions, such as phomoxanthone A. In addition to these interactions, it is worth mentioning hydrogen bonds with Ser80, of the Pi-Alkyl type with Ala61 and Pi-Anion with Glu57 (Figure 3).

**Figure 4:**
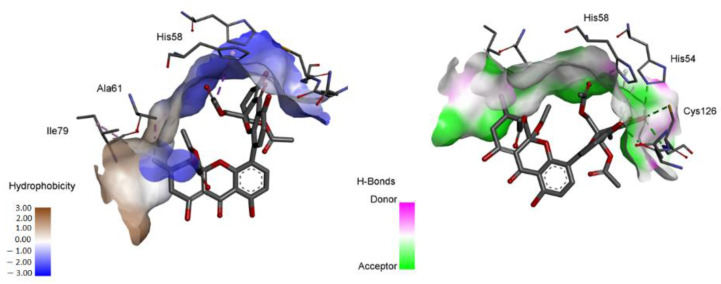
Diagram of hydrophobic surfaces and hydrogen bonds between phomoxanthone A and LuxS (PD ID 2FQO). The figure was generated by the Biovia Discovery Studio Visualizer software (v19.1.0.18287, BIOVIA, San Diego, CA, USA).

Therefore, based on the observation of the interactions of phomoxanthone A with LuxS, it is possible to observe a possible modulation in the activity of the enzyme, since the metal ion (Fe^2+^) is tetrahedrally coordinated by the side chains of histidines 54 and 58 in the motif and Cys126 (numbered according to *B. subtilis*). The interaction with two residues of this catalytic site can lead to the loss of activity [21]. In order to complete the analysis, it is possible to reiterate from the hydrophobic surface and hydrogen bond diagrams (Figure 4) that phomoxanthone A has effective interactions with the catalytic triad residues (His54, His 58 and Cys126). Hydrogen bonds and the hydrophobic surface play a vital role in forming ligand binding specificity with the receptor in the process of drug design in the chemical and biological system, molecular identification and biological activity.

#### 2.4.2. Quantum Studies

In general, structure–activity relationship studies consider the energies of HOMO and LUMO. The reason for this is related to the fact that these properties provide information about the electron-donor and/or electron-acceptor character of a compound and, consequently, the formation of a charge transfer complex (CTC) [22]. The HOMO energy measures the electron-donating character of a compound and the LUMO energy measures the electron-accepting character [23]. From these definitions, two important characteristics can be observed: the higher the HOMO energy, the greater the electron-donating capacity, and the lower the LUMO energy, the lower the resistance to accept electrons. HOMO and LUMO energies have been used for some decades as indices of chemical reactivity and are commonly correlated with other indices, such as electron affinity and ionization potential [24].

**Figure 5:**
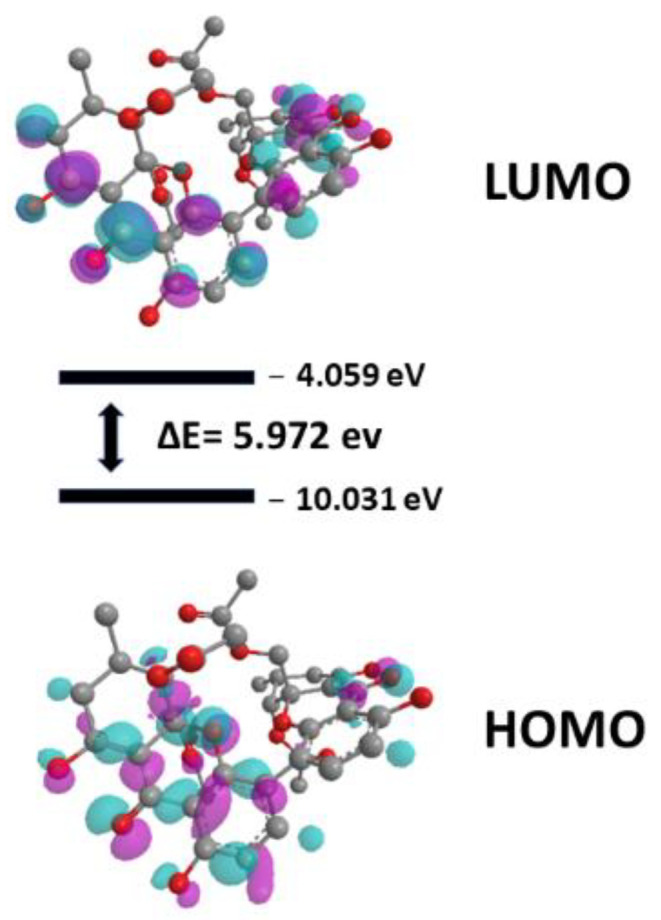
Surfaces of HOMO, LUMO and gap value to phomoxanthone A. In purple, the positive contributions and in cyan blue the negative contributions to the orbitals.

To understand the activity of phomoxanthone A against *B. subtilis*, the surfaces of the HOMO and LUMO orbitals were analyzed, as well as the value of the gap (energy difference between LUMO and HOMO), as can be seen in Figure 5.

## 3. Discussion

The chemical study of the ethyl acetate extract of the endophytic fungus *Paecylomices* sp. EJC01.1 led to the isolation of three xanthone dimers, identified as phomoxanthone A (**1**), phomoxanthone B (**2**) [10] and dicerandrol B (**3**) [18]. This is the first occurrence of phomoxanthone dimers from genus *Paecilomyces*.

Previous studies have shown that phomoxanthones A and B exhibited significant activity against *Mycobacterium tuberculosis*, *Plasmodium falciparum*, *Bacillus megaterium* and fungicidal activity against the *Pyricularia oryzae,* besides having cytotoxic activity against cancer cells [10,25]. Dicerandrol B shows significant activity against *Xanthomonas oryzae* [18]. The secalonic acid D is a correlate compound of phomoxanthone A, phomoxanthone B and dicerandrol B that was isolated of the endophytic fungus *Paecilomyces* sp. by Guo et al. [26]; this enables us to propose that *Paecilomyces* fungi possess enzymes in their biosynthetic route, leading to the formation of dimeric xanthones.

The ethyl acetate extract was the most activity with better result against *E. coli* and *B. subtilis*, when compared to the hexane and methanol extracts from *Paecilomyces* sp. EJC01.1. Close data are found in the evaluation of ethyl acetate extracts of isolate RGM-02 in a study by Radji et al. [27].

Phomoxanthone A (**1**) was active with a bactericidal effect against *B. subtilis* and inhibited the growth of *E. coli* and *S. aureus*, presenting a bacteriostatic effect, which can be compared to polyketides isolated from *Paecilomyces variotii* that also presented significant activity in front of *S. aureus* and other bacteria [28,29].

Endophytic fungi are an important reservoir of therapeutically active compounds. Two dimeric tetrahydroxanthones, phomoxanthone A and 12-*O*-deacetyl-phomoxanthone A, were obtained from the rice culture of *Phomopsis* sp. The activities of these phomoxanthone A and 12-*O*-deacetyl-phomoxanthone A compounds against gram-positive and gram-negative bacteria and fungi strains were evaluated using the agar diffusion method. These compounds exhibited moderate activity against *Botrytis cinerea*, *Sclerotinia sclerotiorum*, *Diaporthe medusaea* and *S. aureus*. However, the compounds lacked activity against the gram-negative bacteria *Pseudomonas aeruginosa* [30]. The crude ethyl acetate extract obtained from *Phomopsis* sp. GJJM07, isolated as an endophyte from the medicinal plant *Mesua ferrea*, was evaluated for antimicrobial and free radical scavenging (DPPH) activity. This extract showed good inhibition against test pathogens, such as *E. coli*, *K. pneumoniae*, *B. subtilis*, *M. luteus* and *C. albicans*. These studies corroborate the results obtained in our study (Table 1). In addition, it is also described that phomoxanthone A showed activity against bacteria and phytopathogenic fungi [25], thus reinforcing the hypothesis of Shiono et al. [30], as they determine that the antimicrobial activity of these compounds produced by *Phomopsis* sp. can play an important role in protecting the host plant against degradation and disease caused by pathogens.

In our study, the compound phomoxanthone A was isolated as the majority and tests against *L. amazonensis* and *T. cruzi* showed good antiparasitic activity. In the literature, it was possible to find a study of the biological potential of phomoxanthones A and B isolated from the endophytic fungus *Phomopsis* sp. BCC 1323. These compounds showed significant activity against *Plasmodium falciparum* (K1, multi drug resistant strain) [10]. In addition, a study with phomoxanthone A, derived from the endophytic fungus *Phomopsis longicolla*, determined that this compound causes a strong release of Ca^2+^ from the mitochondria but not from the endoplasmic reticulum. This depolarization inhibits cell respiration and electron transport chain activity, exclusively affecting the internal mitochondrial membrane, leading to crest rupture, release of pro-apoptotic proteins and apoptosis [31]. Therefore, the antiprotozoal potential of phomoxanthone A may be related to the mitochondrial inhibition pathway of these parasites.

However, the values observed for the SI indicated that there was no great selectivity of phomoxanthone A due to the cytotoxicity values observed in our study. The work by Isaka et al. [10] report that the compound phomoxanthone A also showed cytotoxicity for two cancer cell lines (KB, BC-1) and for Vero cells, and that the activity of this compound may be linked to its lipophilicity.

In the antimicrobial assay, we verified that **1** was active up to the lowest concentration tested against *B. subtilis*, which led us to deepen our study on a computational base. Given the results of the antibacterial assays, phomoxanthone A has a higher activity for *B. subtilis* and a less promising result for *S. aureus*, which naturally led us to exclude DNA gyrase as one of the possible targets [32,33,34]. In addition, CYP51 [35,36] was also not considered in the analysis given its relevance to activity in fungi and trypanosomatids, for which we can observe the results presented for leishmanial and trypanocide activity. Therefore, we investigated a possible response to the antibacterial activity whose hypothesis about the molecular mechanisms involved in the activity may be associated with the modulation of S-ribosyl-homocysteine lyase (LuxS), described in more than 500 different bacterial species, being highly conserved, and involved in controlling the expression of more than 400 genes associated with adhesion, movement, toxin production, among others [37,38].

LuxS is a potential target for the development of new therapies, as it is present in a high number of bacteria [39]. Furthermore, the enzyme controls processes, such as biofilm formation and the synthesis of virulence factors through the synthesis of auto-inductor-2 (AI-2). However, it is thought that it may also regulate biofilm formation through AI-2-independent pathways, such as by directly regulating the expression of biofilm-associated genes. It has already been shown in vivo that inhibition of the LuxS pathway decreases the virulence of some pathogenic organisms [40].

Since LuxS catalyzes essential processes for the survival of bacteria, its inhibition will not exert high selective pressure on them [41]. However, inhibition can lead to the accumulation of toxic products and, indeed, lead to a competitive disadvantage that favors the emergence of resistance [39].

Until we can see that the surface of HOMO is concentrated in one of the portions containing the three rings of phomoxanthone A, with LUMO being more distributed. Analyzing the results of the surfaces, we can observe that there is a small energy value between the HOMO and the LUMO, that is, there is a low value (5.972) for the gap [42]. Overall, the HOMO-LUMO gap of molecules indicates the chemical stability and chemical reactivity, as well as the biological activity that drugs has to find.

Taken together, these results provide evidence that phomoxanthone A has an interesting antibacterial potential, which can be understood from a possible modulation of LuxS activity, as well as from the reactivity profile obtained by analyzing the boundary orbitals and their energy gap.

## 4. Materials and Methods

### 4.1. Fungus

*Paecilomyces* sp. was obtained from a collection of the Laboratório de Bioensaios e Química de Micro-organismos (LaBQuiM), Programa de Pós-Graduação em Química—Universidade Federal do Pará. One strain is deposited in the LaBQuiM with the code EJC01.1 and its identification was based on phenotypic by microscopy optical analyses [43]. This collection contains isolated from *Schnella splendens* (Kunth) Benth (exsiccata nº 177.179).

### 4.2. Bacteria

Test microorganisms were *Bacillus subtilis* (ATCC 6633), *Escherichia coli* (ATCC 25922), *Staphylococcus aureus* (ATCC 25923), *Salmonella typhimurium* (ATCC14028) and *Pseudomonas aeruginosa* (ATCC 27853), which were obtained from Instituto Evandro Chagas, Belém, PA, Brazil.

### 4.3. Parasites

The Y strain of *Trypanosoma cruzi* had been isolated from a patient in the acute phase of Chagas’ disease (Pereira da Silva and Nussenzweig, 1953). The epimastigote forms were cultured at 28 °C in liver infusion tryptose (LIT) medium. The cultures used had a maximum of six in vitro passages. Promastigote forms of *Leishmania amazonensis* H21 (MHOM/BR/76/MA-76) were cultured at 26 °C in Schneider’s insect medium (Sigma-St. Louis, MO, USA) for a maximum of ten in vitro passages. LIT and Schneider’s medium were supplemented with 10% fetal bovine serum (Gibco, Gaithersburg, MD, USA), 100 IU mL^−1^ of penicillin (Gibco, Gaithersburg, MD, USA) and 100 µg mL^−1^ of streptomycin (Sigma, St. Louis, MO, USA).

### 4.4. Animals and Ethical Statements

Healthy 4–6-weeks old female BALB/c mice (20–25 g, *n* = 4), purchased from the Institute of Science and Technology in Biomodels of Oswaldo Cruz Foundation, were used. All procedures performed with these animals were in accordance with the National Council for Control of Animal Experimentation (Conselho Nacional de Controle de Experimentação Animal–CONCEA) and approved by the local Ethics Committee on Animal Care and Utilization (CEUA-IOC L53/2016).

### 4.5. Peritoneal Macrophage Collection and Culture

Peritoneal macrophages were isolated from BALB/c mice administered with 3 mL thioglycolate 3% intraperitoneal for 72 h. Then, cells were cultured overnight, maintained in RPMI 1640 medium and supplemented with 10% fetal bovine sera, plus penicillin (100 U mL^−1^) and streptomycin (100 µg mL^−1^), at 37 °C and 5% CO_2_.

### 4.6. Culture of Paecilomyces sp. in Rice and Chemical Constituents’ Isolation

Twenty-two Erlenmeyer flasks (1000 mL) containing 200 g rice (Tio João^®^) and 125 mL distilled water per flask were autoclaved for 45 min at 121 °C. Small cubes of PDA medium containing mycelium of *Paecilomyces* sp. EJC01.1 were added in 20 Erlenmeyer flasks under sterile condition. Two flasks were used as control. After 30 days of growth at 25 °C, the biomass obtained was macerated with methanol (1) for 24 h to kill the fungus; after this time, the system was filtered given biomass and methanol solution, and the solvent was evaporated on a rotatory evaporator to obtain the methanol extract (1) (MeOH-1 68.78 g). The biomass result was macerated with hexane, ethyl acetate and again methanol (2) (72 h 3× each). The solvents solutions were evaporated under reduced pressure producing hexanic extract 11.39 g (yield of 2.85%), ethyl acetate extract 9.9 g (yield of 0.25%) and methanol extract (2) (MeOH-2 10.2 g, yield of 0.26%). The ethyl acetate extract displayed better result against *E. coli* and *B. subtilis* then the other extracts, and then it was fractionated on silica gel column chromatography elude with hexane, ethyl acetate and methanol in increasing polarity gradient given fractions hexane (F1), hexane/ethyl acetate 1:1 (F2), hexane/ethyl acetate 3:7 (F3), ethyl acetate (F4) and methanol (F5). Fraction F2 was successive fractionations on silica gel column chromatography elude with hexane, ethyl acetate and methanol in increasing polarity gradient were obtained the compounds phomoxanthone A (**1**) (59.5 mg), phomoxanthone B (**2**) (5.0 mg) and dicerandrol B (**3**) (4.0 mg).

### 4.7. General Procedures

Mass spectra (ESIMS) data were acquired using a Waters Acquity TQD instrument. Resonance magnetic nuclear 1D and 2D spectra were recorded on a Varian Mercury 300, using solvent signal (d-chloroform) as reference. The chemical shifts are given in delta (δ) values and the coupling constants (J) in Hertz (Hz).

### 4.8. Antimicrobial Assay

These assays were performed by applying the broth microdilution method, according to the standards described by the Clinical and Laboratory Standards Institute [44]. In 96 well plates, we added 100 μL of culture medium brain heart infusion (BHI) (Himedia), 100 μL of test material and 5 μL of test bacteria at 1.0 × 10^4^ CFU mL^−1^, which were incubated at 37 °C (24 h). The extracts and compound **1** obtained from the fungal culture were dissolved initially 1 mg to compound **1** and 5 mg to extracts in 100 uL of dimethylsulfoxide and 900 μL of BHI broth given 1 mg mL^−1^ and 5 mg mL^−1^ for stock solution. The stock solution was diluted at 500 μg mL^−1^ to 7.81 μg mL^−1^ to compound 1 and 2500 μg mL^−1^ to 39.06 μg mL^−1^ for extracts concentration for the test. Bioactivity was recorded as absence of red coloration in the wells after addition of 10 μL 2,3,5-triphenyltetrazolium chloride. The microorganisms were then sub-cultured on BHI plates. The activities of test compounds were classified as bacteriostatic or bactericidal effects according to the behavior of the microorganisms in these sub-cultures. Penicillin, vancomycin and tetracycline were used as positive controls; BHI culture medium was used as negative control.

### 4.9. Antileishmanial and Antitrypanosomal Activity Assay

The promastigote forms of *L. amazonensis* and the epimastigote forms of *T. cruzi* (2 × 10^6^ parasites mL^−1^) from a 3–5-day-old culture were incubated, for 24 hours, in 96-well plates in the presence of different concentrations of phomoxanthone A (7.81–500 μg mL^−1^), in a final volume of 100 μL per well. Wells without parasites were used as blank and wells with only parasites were used as control. The viability of parasites was evaluated after treatment by counting the total number of live promastigotes, taking into account the flagellar motility, using Neubauer’s camera and optical light microscope. This count was compared with the score of non-treated promastigote growth. This experiment was carried out in triplicate. The results were expressed as parasite growth inhibitory concentration (IC_50_). Amphotericin B (0.016–1.0 μg mL^−1^) and benznidazole (3.125–100 μg mL^−1^) were used as reference drugs.

### 4.10. Cytotoxicity Assay and Selectivity Index

Peritoneal macrophages were cultured in 96 well plates (5 × 10^5^ cells mL^−1^) with different concentrations of compound 1 (3.9–500 μg mL^−1^), amphotericin B (0.915–50 μg mL^−1^) and benznidazole (3.125–200 μg mL^−1^) up to a final volume of 100 μL per well, at 37 °C and 5% CO_2_. Wells without cells were used as blanks and wells with cells and DMSO 1% only were used as controls. After 24 h, cells viability was evaluated by the modified colorimetric method with tetrazolium-dye 3-(4,5-dimethylthiazol-2-yl)-2,5-diphenyltetrazolium bromide (MTT) [45]. The results were used to calculate the 50% cell cytotoxicity (CC_50_). The selectivity index (SI) was obtained from the ratio of peritoneal macrophages CC_50_ and IC_50_.

### 4.11. Molecular Docking

Molecular docking calculations were performed by the GOLD v.2022.1 program, which is available free of charge from CSDS (bdec.dotlib.com.br/inicio_csds/application/Hermes (accessed on 8 August 2022)). In total, 4 different scoring functions (ASP, ChemScore, GoldScore and ChemPLP) were used in the enzyme–substrate interaction calculations. The ligands were treated as flexible and the protein structure (PDB ID 2FQO) as rigid. The determination of the active site was made from the geometric center of the co-crystallized ligand, generating a spherical grid with a radius of 6 Å. To reduce the computational cost, the remaining waters of the enzyme were excluded. The validation process of the applied calculation was carried out through redocking studies, evaluating the RMSD value of the cocrystallized ligand pose that presented the best score value and its alignment between the generated poses. The visual analysis of the results was performed with the help of Discovery Studio visualizer version v19.1.0.18287 (BIOVIA, San Diego, CA, USA) and Maestro v.11.8 software (Schrödinger, LLC, New York, NY, USA). The 2D and 3D enzyme–substrate interaction diagrams were produced in Discovery Studio Visualizer v19.1.0.18287.

### 4.12. DFT Studies

The calculations of quantum studies (DFT) were performed to estimate all energy values of highest occupied molecular orbital (HOMO) and lowest unoccupied molecular orbital (LUMO) and gap (LUMO-HOMO) were performed on Gaussian v.09 program package (Gaussian, Inc., Wallingford, CT, USA) with B3LYP level and 6–311++G(d, p) basis sets [46].

### 4.13. Statistical Analysis

The numerical results were expressed as mean ± standard deviation, and the IC_50_ and CC_50_ were obtained from a nonlinear regression fit curve of concentration log versus normalized response. The statistical analyses were conducted using the statistical software GraphPad Prism^®^ version 7 (GraphPad Software Inc., San Diego, CA, USA) and differences were considered significant when *p* < 0.05.

## 5. Conclusions

The chemical investigation of *Paecilomyces* sp. EJC01.1 resulted in the isolation of three compounds phomoxanthone A, phomoxanthone B and dicerandrol B, which is being reported for the first time in the genus *Paecilomyces*. The extracts were tested against the bacteria and the ethyl acetate extract was most active. The phomoxanthone A, a major compound isolated of ethyl acetate extract, presented good antimicrobial activity against *B. subtilis* and a pronounced inhibitory effect was obtained against *L. amazonensis* and *T. cruzi*. Molecular docking studies signaled a possible antibacterial action pathway against *B. subtilis* by modulating LuxS activity. DFT data demonstrated to a small energy gap that corroborates the activity observed in the experiments. Therefore, it is believed that phomoxanthone A is the chemical marker responsible for the observed biological activity.

## Figures and Tables

**Table 1:** Antimicrobial activity of extracts and compounds isolated from *Paecilomyces* sp. EJC01.1.

Compound	MIC (µg mL^−1^)				
	Gram (+) Bacteria		Gram (−) Bacteria		
	*Bacillus* *subtilis* (ATCC 6633)	*Staphylococcus* *aureus* (ATCC 29213)	*Escherichia* *coli* (ATCC 25922)	*Pseudomonas* *aeruginosa* (ATCC 27853)	*Salmonella* *typhimurium* (ATCC 14028)
Extract MeOH-1	(=) 156.25; (−) 39.06	NT	(=) 312.5; (−) 78.12	(−) 78.12	NT
Extract hexane	(−) 312.5	NT	(−) 312.50	(−) 625	NT
Extract AcOEt	(=) 312.5; (−) 39.06	NT	(=) 1250; (−) 39.06	(−) 312.5	NT
Extract MeOH-2	(=) 625; (−) 78.12	NT	(=) 1250; (−) 312.5	(−) 625	NT
Phomoxanthone A	(=) 7.81	(−) 500	(−) 125	(+)	(+)
Penicillin	(=) 7.81	(=) 7.81	(=) 7.81	(=) 7.81	(=) 7.81
Vancomycin	(=) 7.81	(=) 7.81	(=) 7.81	(=) 7.81	(=) 7.81
Tetracycline	(=) 7.81	(=) 7.81	(=) 7.81	(=) 7.81	(=) 7.81

(+) No activity; (=) bactericidal; (−) bacteriostatic; and (NT) not tested.

**Table 2:** Evaluation of the antileishmanial and trypanocidal activity of substances isolated from *Paecilomyces* sp. EJC01.1.

Compound	Peritoneal Macrophages	*L. amazonensis*		*T. cruzi*	
	CC_50_ (µg mL^−1^)	IC_50_ (µg mL^−1^)	SI	IC_50_ (µg mL^−1^)	SI
Phomoxanthone A	28.79 ± 1.261	16.38 ± 1.079	1.76	28.61 ± 1.071	1.01
Amphotericin B	8.505 ± 1.082	0.02476 ± 1.137	343.5	-	nd
Benznidazole	172.0 ± 1.214	-	Nd	3.459 ± 1.103	49.7

Data represents mean ± SD of at least three experiments realized in triplicate. CC_50_: cytotoxic concentration for 50% of cells; IC_50_: inhibitory concentration for 50% of parasites; SI: selectivity index in relation to cytotoxicity for the BALB/c peritoneal macrophage; nd: not determined.

## Data Availability

Not applicable.
